# Protein Kinase C-Dependent Signaling Controls the Midgut Epithelial Barrier to Malaria Parasite Infection in Anopheline Mosquitoes

**DOI:** 10.1371/journal.pone.0076535

**Published:** 2013-10-11

**Authors:** Nazzy Pakpour, Lauren Camp, Hannah M. Smithers, Bo Wang, Zhijian Tu, Steven A. Nadler, Shirley Luckhart

**Affiliations:** 1 Department of Medical Microbiology and Immunology, School of Medicine, University of California Davis, Davis, California, United States of America; 2 Department of Entomology and Nematology, University of California Davis, Davis, California, United States of America; 3 Department of Biochemistry, Virginia Polytechnic Institute and State University, Blacksburg, Virginia, United States of America; Universidade Federal do Rio de Janeiro, Brazil

## Abstract

Anopheline mosquitoes are the primary vectors of parasites in the genus *Plasmodium,* the causative agents of malaria. Malaria parasites undergo a series of complex transformations upon ingestion by the mosquito host. During this process, the physical barrier of the midgut epithelium, along with innate immune defenses, functionally restrict parasite development. Although these defenses have been studied for some time, the regulatory factors that control them are poorly understood. The protein kinase C (PKC) gene family consists of serine/threonine kinases that serve as central signaling molecules and regulators of a broad spectrum of cellular processes including epithelial barrier function and immunity. Indeed, PKCs are highly conserved, ranging from 7 isoforms in *Drosophila* to 16 isoforms in mammals, yet none have been identified in mosquitoes. Despite conservation of the PKC gene family and their potential as targets for transmission-blocking strategies for malaria, no direct connections between PKCs, the mosquito immune response or epithelial barrier integrity are known. Here, we identify and characterize six PKC gene family members – PKCδ, PKCε, PKCζ, PKD, PKN, and an indeterminate conventional PKC − in *Anopheles gambiae* and *Anopheles stephensi*. Sequence and phylogenetic analyses of the anopheline PKCs support most subfamily assignments. All six PKCs are expressed in the midgut epithelia of *A. gambiae* and *A. stephensi* post-blood feeding, indicating availability for signaling in a tissue that is critical for malaria parasite development. Although inhibition of PKC enzymatic activity decreased NF-κB-regulated anti-microbial peptide expression in mosquito cells *in vitro*, PKC inhibition had no effect on expression of a panel of immune genes in the midgut epithelium *in vivo*. PKC inhibition did, however, significantly increase midgut barrier integrity and decrease development of *P. falciparum* oocysts in *A. stephensi*, suggesting that PKC-dependent signaling is a negative regulator of epithelial barrier function and a potential new target for transmission-blocking strategies.

## Introduction

The protein kinase C (PKC) gene family plays a significant role in eukaryotic cellular differentiation, activation of signaling cascades and survival [1]. In this context, these serine/threonine kinases regulate a broad spectrum of physiologies including growth, reproduction, and immunity. PKCs are highly conserved in eukaryotes, ranging from seven isoforms in *Drosophila melanogaster* [2], [3] to 16 isoforms in mammals [1], [4]. Based on a generalized structure that includes a conserved carboxy-terminal kinase domain, binding sites for specific activators and several other domain features, PKCs can be assigned to the following subfamilies: conventional (cPKC), novel (nPKC), atypical (aPKC), PKC-like (PKN), and protein kinase D (PKD). PKDs were formerly categorized as atypical PKCμ [4]. The cPKCs (PKCα, PKCβ, PKCγ) are activated by calcium-dependent binding of diacylglycerol (DAG) and phospholipids to conserved C1 and C2 domains, while nPKCs (PKC δ, PKCε, PKCη, PKCθ) lack the C2 domain and consequently do not require calcium for activation. In contrast, aPKCs (PKC ζ, PKCι/λ) and PKNs are allosterically activated through conserved Phox/Bem1 (PB1) and homology region 1 (HR1) domains, respectively. PKDs can be directly activated by DAG or indirectly by other PKCs [4]. Within and among organisms, these domains share conserved features, but play distinct, non-redundant roles in cell signaling responses [1]. Despite this conservation and the essential roles of PKCs in various cell signaling pathways, orthologs of these kinases had not been definitively identified in the African (*Anopheles gambiae*) or Asian (*Anopheles stephensi*) mosquito vectors of malaria.

Annually, there are over 250 million new malaria cases, the majority due to infection with *Plasmodium falciparum* [5]. *Plasmodium* development in anopheline mosquitoes begins with ingestion of blood containing male and female gametocytes that quickly develop into micro- and macrogametes that fuse to form mobile ookinetes that penetrate the midgut epithelium 24–32 hours after infection. After growth and development as vegetative oocysts for 10–12 days, thousands of sporozoites are released into the hemolymph, the open circulatory system of the mosquito. These sporozoites invade the salivary glands, where they are released into the saliva and injected into a human host with subsequent blood feeding. The physical barrier of the midgut epithelium, along with the innate anti-parasite defenses of the mosquito, creates a bottleneck for parasite development. Indeed, studies have shown that fewer than 1% of ookinetes formed in the mosquito midgut successfully transition to oocysts [6]. Given the importance of PKC regulation of immune responses and epithelial integrity in mammals and *D. melanogaster* [1], manipulation of PKC signaling could alter the susceptibility of mosquitoes to malaria infection.

For successful infection, ookinetes must traverse the midgut by migration through or between midgut epithelial cells [7]. The midgut is a single monolayer of epithelial cells arranged as an inner apical surface with microvilli and a basal outer surface [8] connected through septate junctions that are similar to mammalian tight junctions [9]. PKCs regulate epithelial barrier function in mammals via the assembly and disassembly of tight junctions [10], [11]. Specifically, aPKCs are important regulators of occludins, integral membrane proteins of mammalian tight junctions [10]. Analogously, PKCs are important regulators of the *Drosophila* orthologs of septate junction occludins known as discs-large-1 tumor suppressors [12], [13]. Based on these observations, we hypothesize that PKCs regulate the midgut epithelial barrier in anopheline mosquitoes, perhaps via modification of septate junctions, to control malaria parasite development.

Prior to and during invasion of the midgut epithelium, ookinetes also encounter mosquito immune defenses that are regulated in part by NF-κB transcription factors [14]. There are five NF-κB isoforms in mammals, three in *Drosophila*, and two (Rel1 and Rel2) in *Anopheles* mosquitoes [15]. NF-κB binding motifs are found in the upstream regions of many *Anopheles* immune genes and Rel1 and Rel2 control mosquito immune responses to bacterial, fungal and parasitic pathogens [14]. Indeed, increased NF-κB-dependent transcription can reduce both bacterial load and *Plasmodium* development in anopheline mosquitoes [14], [16]. PKCs are key regulators of NF-κB transcription factors in mammals [17]. For example, PKCθ is an important mediator of NF-κB-dependent T cell receptor activation [18]. PKCζ is critical for LPS-induced activation of NF-κB in mammalian monocytes and macrophages [19], while *Drosophila* aPKC is required for Toll signaling-dependent activation of NF-κB and the production of antimicrobial peptides (AMPs) [20]. NF-κB transcription factors are also involved in the regulation of epithelial barrier integrity [21]. For example, PKCζ regulation of NF-κB activation contributes to tight junction integrity and endothelial permeability in mammals [22]. Therefore, anopheline PKC-dependent regulation of NF-κB-dependent immune responses and epithelial barrier function is likely to occur during parasite infection.

Herein, we present the identification and characterization of six PKC gene family members in *A. gambiae* and *A. stephensi*. Sequence and phylogenetic analyses of the anopheline PKCs confirmed most subfamily assignments. All six PKCs are expressed in the midgut epithelia of *A. gambiae* and *A. stephensi*, indicating availability for signaling in a tissue that is critical for malaria parasite development. In immortalized anopheline cells *in vitro*, inhibition of PKC enzymatic activity decreased NF-κB-regulated AMP expression in response to bacterial pathogen-associated molecular patterns (PAMPs) and to *P. falciparum* soluble proteins (PfsPs). Although PKC activity positively regulated NF-κB activity *in vitro*, inhibition of PKC activity in *A. stephensi* did not alter immune gene expression in the midgut in response to *P. falciparum* stimuli. However, decreased PKC activity resulted in a significant increase in midgut barrier integrity and significantly decreased *P. falciparum* development in *A. stephensi*, suggesting that PKC-dependent changes to the epithelial barrier are critical to successful malaria parasite development. Therefore, inhibition of PKC signaling could be used in genetic or chemical strategies to disrupt parasite development and transmission.

## Results

### Identification and Characterization of PKC Orthologs in Anopheline Mosquitoes

**Figure 1:**
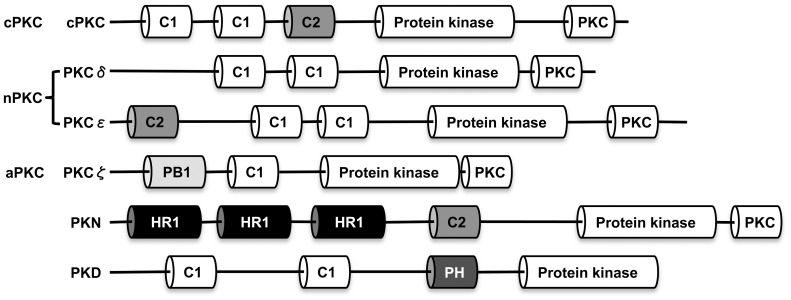
The domain structure of the PKC gene family in *Anopheles gambiae* and *Anopheles stephensi*. Organization of the conserved domains in *A. gambiae* and *A. stephensi* PKC gene family. Based on their regulatory domains, PKC family members can be divided into five structurally and functionally distinct subgroups: classical PKCs (cPKC), novel PKCs (nPKC), atypical PKCs (aPKC), PKC-related kinases (PKN) and protein kinase Ds (PKD). Zinc finger-like cysteine-rich motifs (C1) can function to bind diacylglycerol and phospholipids. C2 domains bind phospholipids. Phox/Bem domain 1 (PB1) functions as a dimerization domain. Homology region 1 (HR1) binds small-GTPases and pleckstrin homology (PH) domains bind membrane lipids and can tether PKCs to other proteins.

Analyses of annotated and unannotated genome sequence data were used to identify six *A. gambiae* PKC gene family members: cPKC, PKCδ, PKCε, PKCζ, PKD, PKN (Table 1, Figure 1). Newly identified PKC genes were further classified into subfamilies (conventional, atypical, novel, PKD, PKN) based on their domain structure (Figure 1) and sequence similarity to PKC-encoding genes from *Caenorhabditis elegans*, *D. melanogaster*, *Danio rerio*, *Xenopus laevis*, *Mus musculus*, and *Homo sapiens* (Table S1). Alignments with published sequences from the aforementioned species revealed predicted phosphorylation sites required for PKC catalytic function in the protein kinase and PKC terminal domains (Table 1) [1].

**Table 1:** PKC gene family members in *A. gambiae* and *A. stephensi*.

*Anopheles gambiae*				
Isoform	Subfamily	Accession number/Ensembl ID	Residues	Predicted phosphorylation sites
conventional	conventional	EAA00266.4/AGAP012252	670	T503, T641, T654
delta δ	novel	EDO64332.2/AGAP000418	614	T443, T576, T583
epsilon ε	novel	EAA07888.5/AGAP002748	778	T598, T745, T766
zeta ζ	atypical	EAA00497.3/AGAP011993 & EAA00702.3/AGAP011988	482	T322, T450, n/d
PKD	protein kinase D	EAA06222.6/AGAP000070	765	S625, S769, T633
PKN	protein kinase C-like	EAA03911.6/AGAP007587	998	T834, T972, T992
***Anopheles stephensi***				
**Isoform**	**Subfamily**	**Accession number**	**Residues**	**Predicted phosphorylation sites**
conventional	conventional	KC896830	662	T495, T633, T646
delta δ	novel	KC896831	611	T443, T576, T583
epsilon ε	novel	KC896832	777	T599, T744, T765
zeta ζ	atypical	KC896833	482	T322, T449, n/d
PKD	protein kinase D	KC896834	770	S622, S626, T630
PKN	protein kinase C-like	KC896835	996	T832, T970, T1286

**Figure 2:**
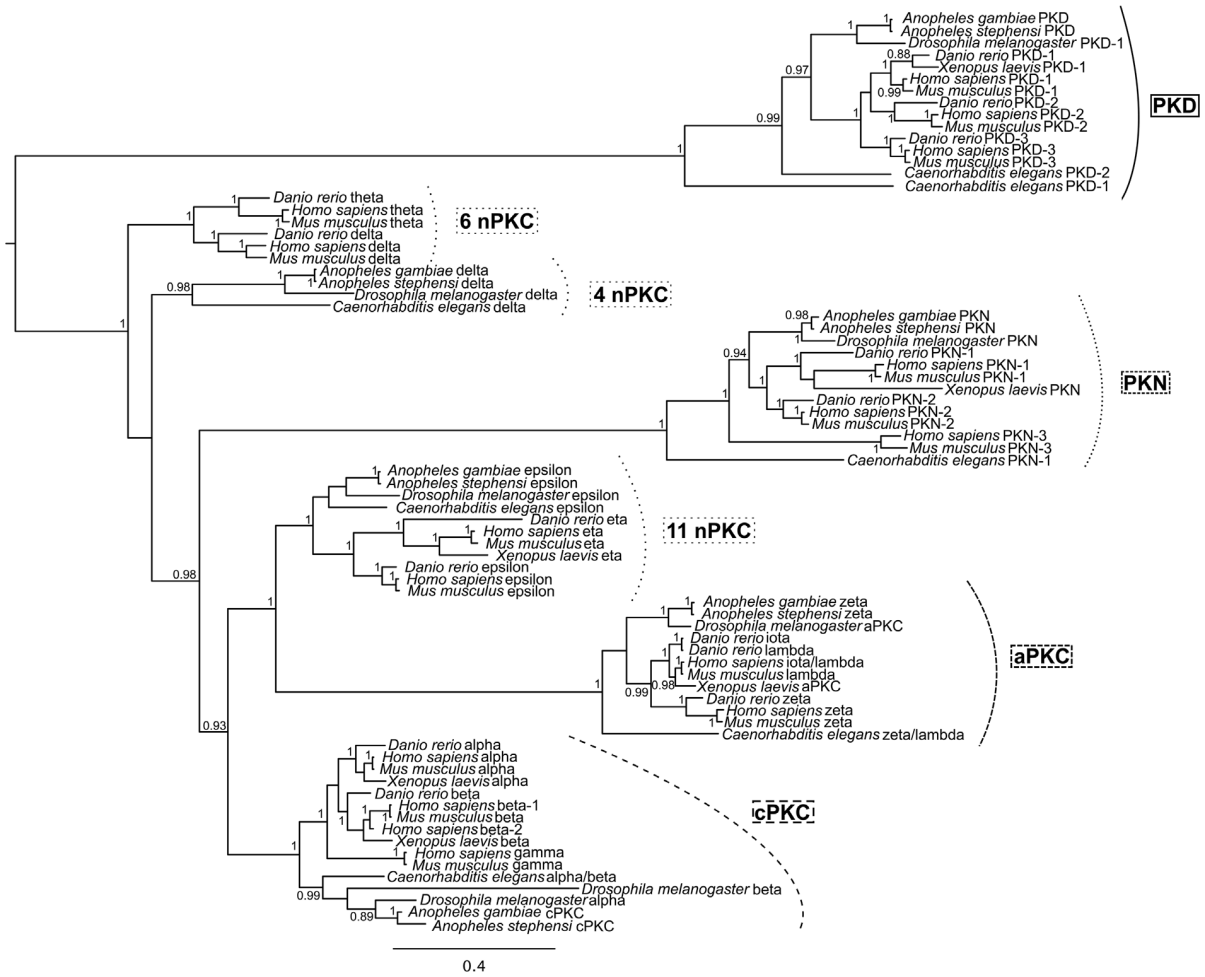
Midpoint-rooted Bayesian tree based on analysis of the GBFILT dataset. Node support values represent Bayesian posterior probabilities (values below 0.85 are not shown). Groupings of specific PKC gene family members are marked. Accession numbers of all sequences used for this analysis are listed in Table S1.

*Anopheles gambiae* cPKC (AGAP012252) was identified based upon the presence of N-terminal C1 and C2 domains common to all cPKCs [1]. Phylogenetic analyses (Figure 2 and S2) placed this gene product within a group of sequences that included α, β, α/β, and γ cPKCs from different organisms. Two nPKCs, PKCδ (AGAP000418) and PKCε (AGAP002748), were identified in *A. gambiae* based on identity to their human counterparts. In both cases, phylogenetic analyses grouped δ and ε *Anopheles* PKCs with similar invertebrate (ecdysozoan) sequences, although the sampled PKCδ sequences did not form a monophyletic group, a result also observed for PKCε sequences (Figure 2). On the negative strand of chromosome 3L, AGAP011993 and AGAP011988 were found to encode aPKC PB1 and PKC domains, respectively. Upon further analysis, these DNA regions were found to be separated by introns corresponding to the remaining aPKC C1 and protein kinase domains. Alignments with published sequences suggested that AGAP011993 and AGAP011988 were derived from a single *A. gambiae* PKCζ gene. Detection of a protein of the size expected for PKCζ with phospho-specific antisera (based on a mammalian peptide with 93% identity to the predicted mosquito sequences) in both *A. stephensi* and *A. gambiae* immortalized cells (Figure S1) provided additional confirmation of our annotation. The presence of an encoded HR1 domain, together with protein kinase and PKC domains, identified AGAP007587 as *A. gambiae* PKN. Similarly, the presence of a pleckstrin homology (PH) domain along with C1 and protein kinase domains led us to predict that AGAP000070 encoded an *A. gambiae* PKD. PKDs can be difficult to identify based solely on the PH domain, which is common to many proteins that signal by protein interaction [23], therefore a constellation of common C1 and protein kinase domains without a PKC domain is necessary for identification. To support our annotation of PKD, we used a strategy similar to that for PKCζ above to identify a phospho-protein of the expected size in *A. stephensi* and *A. gambiae* cells (Figure S1). All *A. stephensi* PKC genes were identified in the preliminary annotation of *A. stephensi* genome sequence based upon their homology and conserved exon-intron organization (Figure S3) to *A. gambiae* PKC sequences (Table 1, Figure 1).

### Phylogenetic Analysis of Anopheline PKCs

Two datasets, termed UNFILT and GBFILT, were used to infer phylogenetic relationships. The UNFILT dataset included all 1785 amino acid characters following alignment with ClustalX [24] and the GBFILT dataset included 397 alignment sites that were retained after using GBlocks [25] to exclude ambiguously aligned positions. Topologies for the Bayesian consensus trees were similar for both the GBFILT (Figure 2) and UNFILT (Figure S2) datasets, defining the same main PKC groups in both cases. Both trees are midpoint-rooted because sequences from suitable outgroup species were not available. In trees for both datasets the PKD, PKN, aPKC, and cPKC sequences were each monophyletic with Bayesian posterior probabilities (BPP) of 1.0. The nPKC sequences were not monophyletic in trees from both datasets, and this result was strongly supported (BPP) in both GBFILT and UNFILT trees. The ε and η nPKCs formed a strongly supported group in analyses of both datasets, and this clade was the sister group to the aPKC clade, with a BPP of 1.0 (Figure 2 and S2). In contrast, the δ and θ nPKCs were not monophyletic in the GBFILT tree; in the UNFILT tree these sequences formed a single group, but without reliable BPP. PKCs for *A. gambiae* and *A. stephensi* were resolved as sister taxa, frequently with high BPP. In addition, within specific PKC groups, *Anopheles* PKCs were sister to *D. melanogaster* sequences in all cases (Figure 2). Similarly, within PKC groups, PKCs from vertebrate hosts were monophyletic with high BPP (Figure 2).

### Expression of PKCs in *A. gambiae* and *A. stephensi*

**Figure 3:**
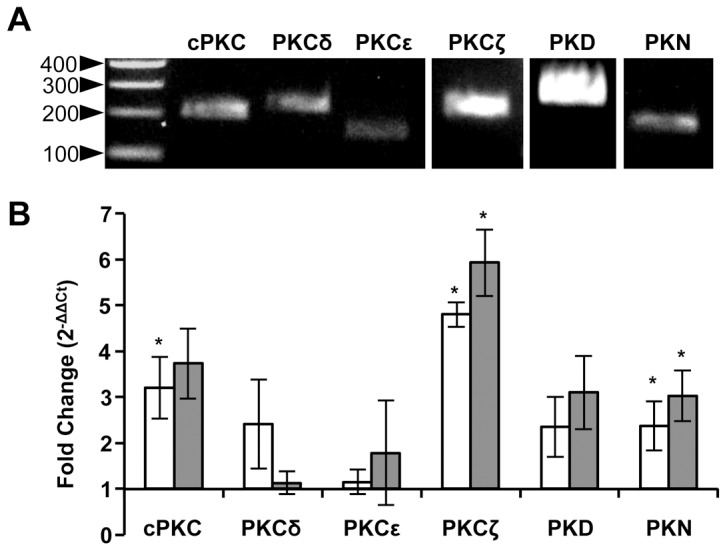
Expression of the PKC gene family in *Anopheles stephensi* midgut epithelium. Total RNA was isolated and converted to cDNA from 30 dissected *A. stephensi* midguts at 24 h post-blood feeding. (A) PKC-specific primers were used to amplify cDNA by conventional PCR, ladder (bp) is shown on left. (B) Fold change in the expression of PKC genes in blood fed (white bars) or FTPP fed (gray bars) mosquitoes compared to non-blood fed control mosquitoes as determined by qRT-PCR. Means ± SEMs from 2–3 independent experiments with separate cohorts of mosquitoes. Pairwise comparisons of treatments and matched controls (non-blood fed *A. stephensi*) were analyzed by Student’s t-test, *p<0.05.

All *A. gambiae* PKCs (Table 1) are expressed in adult female tissues, including the midgut [26]. Interestingly, midgut expression of *A. gambiae* cPKC (AGAP012252) was reported to increase during blood meal digestion [27] and during infection with the mouse malaria parasite *Plasmodium berghei* [28]. In *A. stephensi*, mRNAs for all six PKC-encoding genes were detectable in the midgut at 24 h post-blood feeding by conventional PCR (Figure 3A). To quantify differences in the expression of PKCs in response to blood feeding and to *P. falciparum* freeze/thaw parasite products (FTPP), we also examined midgut tissues 24 h post-blood feeding by real-time PCR. Blood feeding induced significant increases in mRNA expression levels of cPKC, PKCζ, and PKN compared to non-blood fed controls (Figure 3B), with PKCζ being the most highly expressed. While Increases in mRNA expression in response to blood and FTPP for PKCζ, and PKN were significantly different from control levels, increased expression levels in the two treatments were not significantly different from each other. Of all the PKCs, only PKCδ and PKCε expression were unchanged following feeding with blood or with FTPP (Figure 3B). These data confirm that PKCs are expressed in midgut epithelia of *A. gambiae* and *A. stephensi* in response to blood feeding and that PKC expression is similarly induced by a blood meal with and without *P. falciparum* parasite products.

### Inhibition of PKC Activity Decreases *P. falciparum* Development in *A. stephensi*

Having confirmed that PKCs are differentially expressed in the midgut of *A. stephensi* following blood feeding, we sought to determine the impact of PKC-dependent signaling on *P. falciparum* development in the mosquito. Female *A. stephensi* mosquitoes were provided identical infectious blood meals enriched with *P. falciparum* gametocytes that were supplemented with 1 µM chelerythrine, 0.05 µM Go6983, or an equivalent volume of phosphate-buffered saline (PBS) as a control and the number of oocysts were determined at 10 days post-blood feeding. Two-way ANOVA indicated significant effects of experiment (p<0.05), thus individual replicates were analyzed by Kruskal–Wallis test and Dunn’s post-test. In replicates 1 and 3, we found that mosquitoes fed an infectious blood meal in the presence of either PKC inhibitor had significantly fewer oocysts than control mosquitoes (Table 2). In replicate 2, the same trends were evident, but the reductions in oocyst development were not significantly different from control levels (Table 2). Prevalences of infection (the presence of at least one oocyst in a dissected mosquito) were not significantly different, as determined by chi-squared test, among the control and treatment groups (Table 2). These data suggested that inhibition of PKC-dependent signaling alters mosquito biology in a manner that inhibits malaria parasite development.

**Table 2:** Inhibition of PKC activity reduces *Plasmodium falciparum* oocyst development in *Anopheles stephensi*.

	Buffer			Chelerythrine			Go6983				
Exp	N	mean oocyst±SEM	% inf	N	mean oocyst±SEM	% inf	p-value	N	mean oocyst±SEM	% inf	p-value
**1**	48	2.31±0.28	81.3	47	1.28±0.22	63.8	0.002	49	1.61±0.23	71.0	0.034
**2**	47	2.15±0.28	74.5	49	1.55±0.23	67.3	0.064	49	1.80±0.22	83.7	0.199
**3**	50	7.34±1.15	78.0	49	3.96±0.54	87.8	0.045	50	2.72±0.35	82.0	0.002

**Figure 4:**
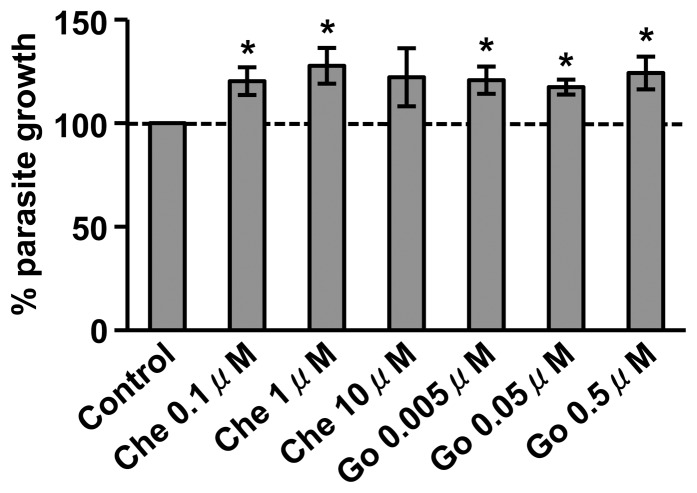
Inhibition of PKC activity increases *Plasmodium falciparum* asexual stage growth *in vitro*. Replicate cultures *of P. falciparum* NF54 were incubated with increasing concentrations of chelerythrine or Go6983. Relative growth is compared to the DMSO control, which is set at 100%. Pairwise comparisons of treatments and matched controls were analyzed by Student’s t-test, *p<0.05.

Although the *P. falciparum* genome does not appear to encode any prototypical PKCs [29], we sought to confirm that the observed effects on oocyst development were due to inhibition of mosquito PKC signaling rather than inhibitor alterations of intrinsic growth of the parasite. To this end, we measured growth of synchronized asexual *P. falciparum* parasites treated with 0.1–10 µM chelerythrine or with 0.005–0.5 µM Go6983 *in vitro*. Interestingly, parasites grown in the presence of either 0.1 µM or 1 µM chelerythrine, and in all concentrations of Go6983, exhibited significantly increased growth at 48 h post-treatment (Figure 4). Although this growth assay cannot be performed efficiently on mosquito-stage parasites, our results suggest that infection patterns observed *in vivo* using the PKC inhibitors Go6983 and Chelerythrine were not due to a direct negative effect on parasite development. Indeed, treatment with the inhibitor resulted in *P. falciparum* growth *in vitro,* not death as observed during parasite development in *A. stephensi in vivo*.

### PKC Activation is Critical for PAMP Induction of NF-κB-dependent Promoter Activity *in vitro*

To determine whether PKC activation in mosquito cells was specific to parasite-derived signals or was more broadly responsive to a variety of pathogen-derived signals, we utilized two prototypical bacterial PAMPs, lipopolysaccharide (LPS) and peptidoglycan (PGN) as well as a soluble preparation of *P. falciparum* proteins (PfsPs) to probe stimulus-specific and PKC-dependent control of NF-κB-dependent promoter activity.

**Figure 5:**
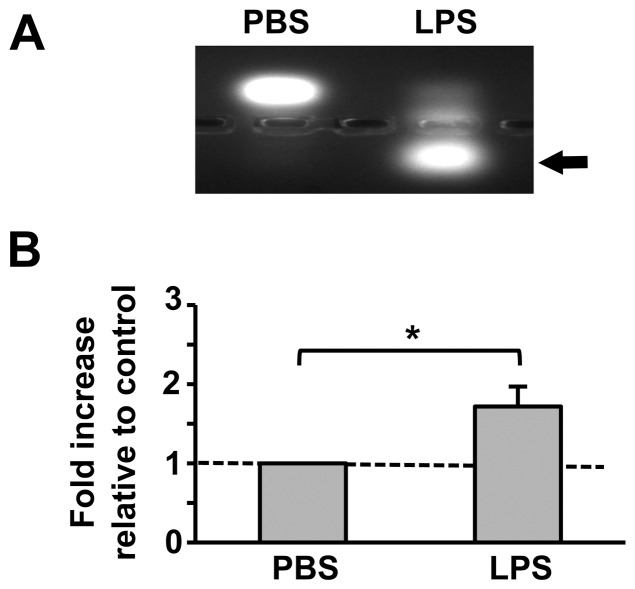
LPS stimulation induces PKC activity in mosquito cells. Cell lysates from immortalized *A. stephensi* ASE cells stimulated with PBS as a control or with 100 µg/ml LPS for 30 min were incubated with fluorescently tagged C1 peptides (a PKC substrate) for 30 min and analyzed by agarose gel electrophoresis. Phosphorylated C1 peptides, which are directly correlated with PKC enzymatic activity, are indicated by the arrow. (A) Representative agarose gel image. (B) Means ± SEMs of phosphorylated C1 peptide fluorescence normalized to PBS treated controls, n = 4. Pairwise comparisons of treatments and matched controls were analyzed by Student’s t-test, *p<0.05.

LPS has been shown to activate several mammalian PKC isoforms involved in Toll-like receptor signaling and host defense [30], [31]. Therefore, we hypothesized that LPS would activate PKC enzymatic activity in mosquito cells as well. To test this hypothesis, cell lysates from immortalized *A. stephensi* embryonic (ASE) cells, stimulated with either PBS as a control or 100 µg/ml LPS, were incubated with fluorescently tagged C1 peptide (a PKC substrate, Figure 5A). LPS stimulation resulted in a significant increase in phosphorylated C1 compared to controls (Figure 5B), confirming that bacterial PAMPs can induce PKC activation in *A. stephensi* cells in a manner similar to that described in human cells [32].

**Figure 6:**
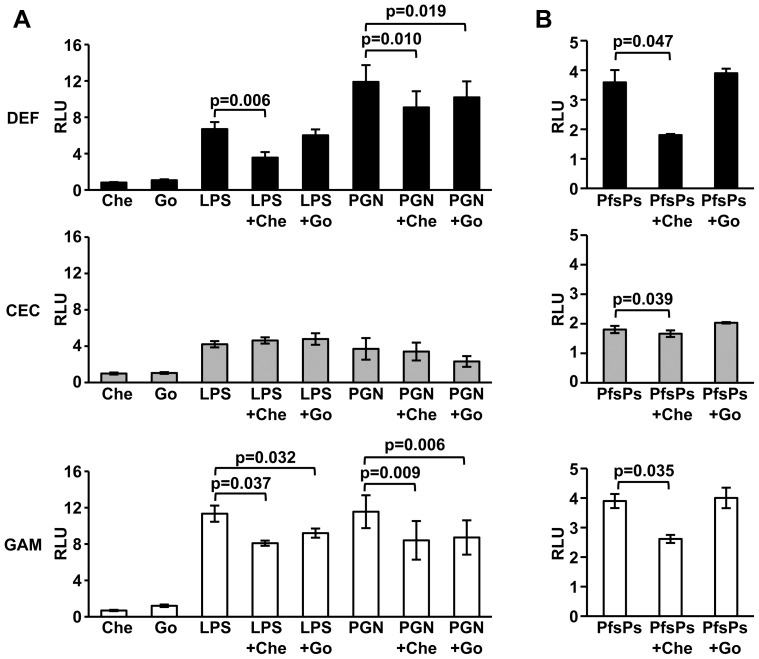
Inhibition of PKC activity decreases antimicrobial promoter activity in immortalized *A. stephensi* cells. ASE cells transfected with Defensin, Cecropin or Gambicin luciferase promoter-reporter plasmid constructs were treated with 1 µM chelerythrine (Che) or 0.05 µM Go6983 (GO) for 1 h prior to stimulation with (A) 100 µg/ml LPS or 1 µg/ml PGN or (B) *P. falciparum* soluble products (PfsPs). Graphs represent means ± SEMs of luciferase activity (relative light units, RLU) normalized to untreated controls, n = 3–12. Pairwise comparisons of treatments and controls were analyzed by Student’s t-test, significant p-values are shown.

To determine whether bacterial PAMP-mediated PKC activation in *A. stephensi* cells was functionally relevant to immune signaling, we utilized a luciferase-reporter assay to quantify NF-κB-dependent AMP promoter activation in ASE cells. For these assays, ∼1.5 kb upstream of the transcriptional start sites for the AMP genes Defensin, Cecropin, and Gambicin were used to construct luciferase reporter plasmids. As expected, treatment of cells with the PKC inhibitors chelerythrine or Go6983 alone did not alter basal activity of any of AMP promoters in ASE cells (Figure 6A). However, inhibition of PKC activity by chelerythrine significantly reduced LPS- and PGN-induced Defensin and Gambicin promoter activity (Figure 6A, top and bottom panels), but had no effect on LPS or PGN-induced Cecropin promoter activity (Figure 6A, middle panel). As previously reported, Cecropin promoter activity was markedly lower then Defensin and Gambicin promoter activities following stimulation (Figure 6A and B) [33], which may limit detection of changes in PKC-dependent signaling. Like chelerythrine, Go6983 significantly reduced PGN-induced Defensin and Gambicin promoter activity and LPS-induced Gambicin promoter activity, but had no significant effect on Cecropin promoter activity (Figure 6A). While both PKC inhibitors resulted in decreased AMP promoter activity following stimulation with bacterial PAMPs (Figure 6A, top and bottom panels), only chelerythrine pre-treatment significantly reduced PfsPs-induced AMP promoter activity (Figure 6B).

### Inhibition of PKC Activation does not Alter *P. falciparum*-induced Expression of NF-κB-dependent Immune Genes *in vivo*

**Figure 7:**
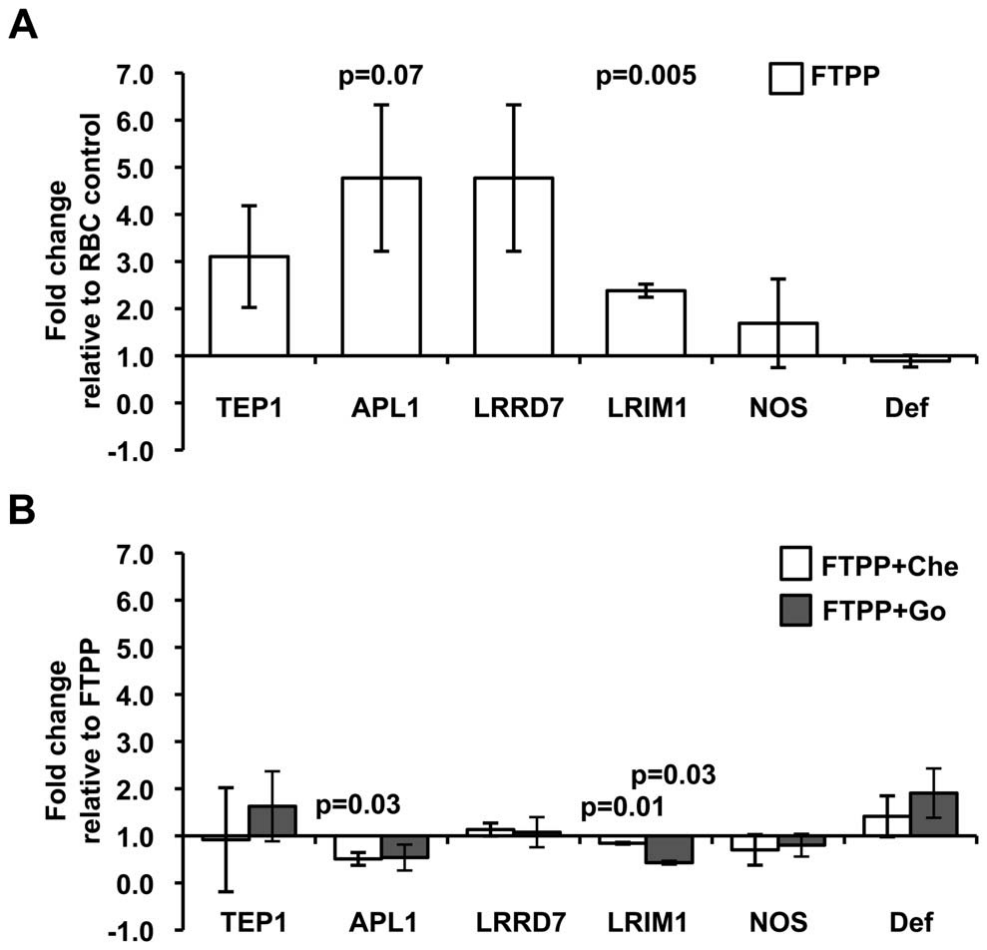
Inhibition of PKC activity does not alter parasite-inducible expression of immune genes in the *A. stephensi* midgut. (A) Fold change in the expression of selected immune genes in the midgut of mosquitoes fed *P. falciparum* freeze/thaw parasite products (FTPP) relative to mosquitoes fed frozen-thawed uninfected RBCs at 24 h post-blood feeding. (B) Fold change in the expression of selected immune genes in the midgut of mosquitoes fed FTPP plus 1 µM chelerythrine (Che) or 0.05 µM Go6983 (GO) relative to mosquitoes fed FTPP alone at 24 h post-blood feeding. Graphs represent means ± SEMs from 3–5 independent experiments. Pairwise comparisons of treatments and matched controls were analyzed by Student’s t-test, p-values are shown.

Our *in vitro* data indicated that PKC activity positively regulates NF-κB-mediated signaling, which suggested that this pathway could contribute to the regulation of parasite development *in vivo*. To test whether PKC-dependent signaling indeed functions in this capacity, we examined expression levels of known anti-parasite immune genes (*nitric oxide synthase* or *NOS, LRIM1, TEP1, APL1, LRRD7*) and an immune gene marker (*defensin*) that are regulated by Rel1-dependent Toll, Rel2-dependent Immune deficiency (IMD) and/or Janus Kinase and Signal Transducer and Activator of Transcription (JAK-STAT, [16], [34]–[39]) in *A. stephensi* following blood meals containing *P. falciparum* freeze/thaw parasite products (FTPP) in the presence or absence of PKC inhibitors. FTPP increased expression of the complement-like glycoprotein *TEP1* and the leucine-rich repeat proteins *LRRD7*, *LRIM1*, and *APL1* in the midgut epithelium at 24 h post-blood feeding (Figure 7A). No inductions relative to controls were observed for *NOS* or *defensin* expression following FTPP feeding (Figure 7A). In contrast to our expectation that inhibition of PKC activity would reduce FTPP-dependent induction of this panel of immune genes, PKC inhibition by chelerythrine or Go6983 had no effect relative to controls (Figure 7B). These data suggested that PKC inhibition did not reduce parasite development (Table 2) via direct activation of immune gene products and/or signaling pathways associated with parasite killing.

### Inhibition of PKC-dependent Signaling Increases Midgut Epithelial Barrier Function in *A. stephensi*

**Figure 8:**
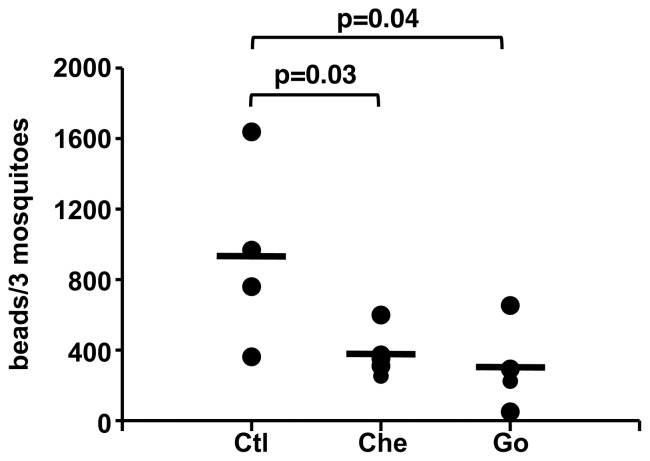
Inhibition of PKC activity increases midgut epithelial barrier integrity in *Anopheles stephensi.* Mosquitoes were fed fluorescent beads (3–3.5 µM) in a blood meal in the presence or absence of PKC inhibitors and, at 72 h post-blood feeding bead numbers were quantified using flow cytometry. Dots represent bead numbers per three whole mosquitoes, means are indicated as bars. Midgut beads averaged 932±267 (mean ± SEM) for blood-fed controls and 377±66 and 305±127 for chelerythrine and Go6983 treated mosquitoes. Pairwise comparisons of treatments and matched controls were analyzed by Student’s t-test, p-values are shown.

PKCs are involved in the regulation of tight junction integrity and polarization of epithelial barriers in mammals [40], [41], phenomena that prevent the diffusion of toxins, allergens, and pathogens from enclosed lumina into tissues [42], [43]. To determine whether PKCs were similarly involved in the regulation of barrier function in mosquitoes, we tested midgut permeability in *A. stephensi* provided with a blood meal containing fluorescently labeled microsphere beads with or without the PKC inhibitors chelerythrine and Go6983. Bead numbers were quantified in midguts or in whole mosquitoes at 72 h post-blood feeding to allow for complete blood meal digestion. Body bead counts (minus midgut beads) in control mosquitoes were 932±267 (mean ± SEM), whereas mosquitoes fed PKC inhibitors had 30–40% fewer beads in the body (chelerythrine 377±66, Go6983 305±127, Figure 8). This significant reduction in the accumulation of beads in the body cavity of mosquitoes (Figure 8) indicates that inhibition of PKC signaling increases midgut epithelial barrier integrity, a response that mirrors PKC-dependent signaling control of epithelial barrier integrity in mammals.

## Discussion

There have been relatively few phylogenetic studies of PKCs [44]–[47] and this is the first analysis to include a wide evolutionary range of species. Here we have identified six novel PKCs − cPKC, PKCδ, PKCε, PKCζ, PKD, PKN − in *A. gambiae* and *A. stephensi* (Table 1, Figure 1). While we were unable to resolve an isoform assignment for the single anopheline cPKC, we are confident of this grouping based on a shared domain structure that defines all cPKCs (Figure 1) and similarity at the amino acid level that exceeds 80% when compared to insect cPKCs from *Bombyx mori* (NP_001036978.1), *Choristoneura fumiferana* (ABZ88709.1), *Apis mellifera* (NP_001128420.1), and *Aedes aegypti* (XP_001652409.1). Further, phylogenetic analyses showed that the anopheline cPKCs are most closely related to *D. melanogaster* and *C. elegans* cPKCs, representing the other sampled ecdysozoans in the analyses (Figure 2 and S2). The Bayesian analyses of amino acid characters recovered distinct monophyletic groups for PKD, PKN, aPKC, and cPKC sequences. Within these four groups, sequences from vertebrates consistently formed a separate sub-clade, whereas ecdysozoan sequences did not always group together, due to the rogue behavior of *C. elegans* sequences (Figure 2, PKD, PKN, and aPKC). The nPKCs did not form a monophyletic group, instead yielding 2–3 separate groups, depending on the phylogenetic dataset. The ε and η nPKCs were strongly supported as the sister group to aPKC sequences, whereas θ and δ nPKCs were more distantly related. These results indicate that sequences currently defined as nPKCs do not share common ancestry, and according to the phylogenetic trees, must represent at least two separate origins not reflected by the current gene nomenclature.

The generalized structure of a conserved kinase domain coupled with a series of regulatory domains defines PKC subfamily classification and dictates some aspects of temporal and spatial activity of these signaling proteins. However, PKCs are regulated by a variety of mechanisms not defined by these features that can limit phylogeny-based interpretations of both activation and function. In particular, PKCs are regulated at the mRNA level and can exhibit distinct tissue- and cell-specific expression patterns [48]. PKCs are also regulated through localization to specific sub-cellular compartments that can facilitate or inhibit their phosphorylation and activation [49], [50]. Finally, PKCs can be regulated by phosphorylation and/or binding of co-factors to regulatory domains [51]. In light of the multiple and overlapping modes of regulation possible for PKCs, analysis of mRNA expression levels provides the most basal measure of PKC activity in mosquitoes. Future studies will focus on determining which PKCs and which PKC-dependent signaling pathways are specifically involved in the regulation of immunity and midgut barrier function in mosquitoes during malaria infection.

The suite of expressed PKCs in the *A. stephensi* midgut at 24 h post-blood feeding (Figure 3A) suggested that PKC activation is temporally consistent with malaria parasite development in the mosquito midgut and that the pattern of expression is not significantly altered in the presence of parasite products (Figure 3B). Indeed, our data confirmed that inhibition of PKC activation significantly reduced *P. falciparum* oocyst development in *A. stephensi* (Table 2). It is unlikely that the observations made in these studies are the result of non-target inhibitor effects as Go6983 and chelerythrine yielded similar results despite the fact that the mechanisms of PKC inhibition and possible non-target effects differ between these two inhibitors.

Specific inhibition of PKCs, however, is clearly expected to be associated with measurable effects on networked downstream signaling proteins [52]–[54]. Hence it is not surprising that we have observed effects of PKC inhibition on phosphorylation of ERK and of FOXO (Figure S4), mediators of pathways that are highly networked with PKC signaling and with NF-kB signaling [55]. For example, PKCs can directly regulate Toll- and Toll-like receptor-dependent immune gene expression (reviewed in [32]), suggesting that inhibition of PKC signaling would enhance expression of immune genes associated with parasite killing. If this were the case, activation of PKC signaling during infection would dampen anti-parasite responses, thereby facilitating parasite development. However, our data did not support involvement of PKC signaling in the regulation of immune gene expression in the midgut (Figure 7). Further examination of the effect of specific PKC isoforms on a broader range of immune effectors may reveal analogous PKC regulation in the mosquito.

An alternative, and perhaps more likely, explanation is that the decrease in expression observed in our AMP promoter-reporter assays following inhibition of PKC activity *in vitro* may be compensated for *in vivo* by signaling pathways that regulate these gene products independently of PKCs. Nevertheless, a contribution of PKC activity to the regulation of NF-kB-dependent signaling in *A. stephensi* cells is clear and consistent with published data from a variety of other organisms, including *D. melanogaster* [17]–[22]. Alternatively, the discordance in our data may reflect true differences between response patterns of mosquito cells *in vitro* to individual stimuli (LPS, PGN, or PfsPs) and response patterns of the highly specialized midgut epithelium to multiple concurrent stimuli in the complex context of blood meal digestion. Mosquitoes ingest 3–10 times their body weight in blood, which is mainly comprised of the protein hemoglobin [56]. The digestion of hemoglobin releases heme, which in turn catalyzes the synthesis of reactive oxygen species (ROS) that at low concentrations function as signaling molecules in pathways involved in growth, differentiation, and immunity [57]. Specifically, ROS can induce PKC activity through the oxidative modification of PKC regulatory domains [58]. In *Ae. aegypti*, heme-induced ROS have been shown to activate PKCs, which allows for the proliferation of intestinal commensal microbiota [59] to 2–3 logs above baseline in the mosquito midgut [60]. The proliferation of commensals may activate PKC-dependent and PKC-independent immune signaling networks to influence the transmission of malaria parasites by mosquitoes [61], [62]. It is possible, therefore, that the decrease in expression observed in our AMP promoter-reporter assays following inhibition of PKC activity *in vitro* is compensated for *in vivo* by additional PKC-independent immune activation signals including ROS or the expansion of the gut microbiota.

PKCs expressed in the midgut epithelium included both conventional and atypical PKCs as well as PKD and PKN. Intriguingly, atypical PKCζ was the most highly expressed following blood feeding (Figure 3B), which is consistent with the role of aPKCs as important regulators of epithelial barrier function in mammals and *Drosophila* [10], [12], [13]. Indeed, PKCζ in particular has been shown to alter tight junctions through the phosphorylation of occludins [22], [40], [63], suggesting that *Anopheles* PKCζ may similarly control epithelial barrier integrity through the regulation septate junctions in the midgut epithelium. In other infection models, the degradation of tight junctions and the subsequent disruption of barrier function have been linked to changes in susceptibility to infection in the mammalian intestine. For example, infections with either *Escherichia coli* or *Giardia lamblia* can disrupt intestinal tight junctions, resulting in loss of barrier function that is associated with increased pathogen colonization [64], [65]. To establish infection in mosquitoes, ookinetes must penetrate the midgut epithelium. Ookinetes are the largest motile life cycle stage of the malaria parasite, measuring 10–12 µm long and 2–3 µm wide [66]. Ookinetes are capable of extensive movement and transverse several epithelial cells before reaching the basal lamina of the mosquito midgut and forming oocysts [7], [67]. Therefore, changes in trans-epithelial resistance of the mosquito midgut could contribute to the success of malaria infection in the mosquito. Indeed, when actin polymerization – a process required for proper functioning of tight junctions [68] – is inhibited in *A. gambiae* the ability of *P. berghei* to invade the midgut increases significantly [28]. These observations, in conjunction with our results (Figure 8), suggest that enhancing epithelial junctions impairs the transit of malaria parasites across the midgut barrier.

While the development of genetically modified mosquitoes could leverage anopheline PKC signaling biology to block transmission in the mosquito host, developments in the use of PKC inhibitors in clinical settings provide an additional relevant practical extension of our work. In particular, PKC-dependent signaling has been targeted aggressively for drug development in humans and selective PKC kinase inhibitors are currently in phase II clinical trials [69], [70]. Mammalian PKCs have also been implicated in the regulation of malaria parasite egress from RBCs and treatment of *P. berghei-*infected mice with the PKC inhibitor sotrastaurin [71], [72] significantly decreased parasitemia and increased survival of infected hosts [73]. Given that circulating levels of sotrastaurin in blood can range from ∼0.1–2.3 µM following a single treatment [74], parasites that ‘escape’ this targeted drug treatment in the human host would be ingested along with the inhibitor by mosquitoes that feed on these treated hosts. Ingested PKC inhibitor – as indicated by our data – can block parasite development in the mosquito host, providing a second level of control under natural conditions for transmission. Therefore, our work indicates that PKC inhibitors that target parasite development in the mammalian host could simultaneously decrease malaria transmission by the mosquito vector, providing an extended activity that has been recently recognized as a critical need for anti-malarial drugs [75].

## Materials and Methods

### Identification and Characterization of *A. gambiae* and *A. stephensi* PKC Gene Family Members

Basic Local Alignment Search Tool (BLAST; [76]) was used to search the translated annotated *A. gambiae* genome sequence with PKC protein sequences from *C. elegans, D. melanogaster, D. rerio, H. sapiens, M. musculus,* and *X. laevis*. Hits with e-values less than 0.01 were analyzed for protein domain structure using Ensembl and the NCBI Conserved Domain Database (CDD). The unannotated *A. gambiae* genome was searched for PKC hidden Markov models (HMM) with HMMER and Pfam using publicly available (www.vectorbase.org) sequence data [77], [78]. Significant BLAST hits were used to validate *A. gambiae* PKCs that had been identified from the annotated genome and to construct the HMM sequences to reveal new *A. gambiae* PKC encoding genes. *A. gambiae* PKC sequences were used to search unannotated *A. stephensi* genome sequence data for PKC gene family members. To identify the full-length coding sequence of the *A. stephensi* PKCs, *A. gambiae* PKC amino acid sequences were used as a query to identify homologous sequence in the June 2010 unpublished draft of the *A. stephensi* assembly by TBLASTN (e-value cutoff 1e-7). The region with the best match and 1-kb flanking sequences were retrieved. Encoded domain sequences and putative phosphorylation sites of PKC-encoding genes were confirmed with ClustalX and manual protein sequence alignments. Putative translational start sites of newly assembled PKC encoding genes were predicted using the ExPASy translate tool (www.expasy.org) and adherence to Kozak consensus [79].

### Phylogenetic Analysis

In total, 77 PKC sequences from eight organisms, including *A. gambiae* and *A. stephensi* (Table S1) were used for phylogenetic analyses. Sequences were aligned in ClustalX v2.1 [24]. Following alignment, poorly aligned positions or highly divergent regions were identified using GBlocks v0.91b [25], with parameters set to allow the least stringent selection. Based on the results from GBlocks, two datasets were created: UNFILT, containing all 1785 initial positions, and GBFILT, containing the 397 positions (22%) retained by GBlocks. ProtTest v2.4 [80] was used to determine the appropriate model of protein evolution for both datasets. Candidate models were restricted to those that can be implemented in MrBayes v3.1.2 [81]. Based on the Bayesian Information Criterion, the models chosen for UNFILT and GBFILT were WAG+G and JTT+I+G, respectively. For consistency, the more complicated model, WAG+G, was implemented for both datasets in subsequent Bayesian analyses. Phylogenetic trees were inferred for each dataset using MrBayes on the CIPRES computing cluster [82]. Four MCMC chains were run for each dataset and both datasets were run for 10×10^6^ generations, with sampling of trees every 10,000 generations. The standard deviation of split frequencies was <0.01 for both datasets at the end of each run. Burn-in was determined empirically and majority-rule consensus trees were visualized in FigTree v1.3.1 (http://tree.bio.ed.ac.uk/software/figtree/). Due to the lack of available sequences from appropriate outgroup species, phylogenetic trees were midpoint rooted. Resulting trees were edited with InkScape 0.48 (http://inkscape.org/).

### Mosquito Rearing and Experimental Treatments

*Anopheles stephensi* Liston (Indian wild-type strain) were reared and maintained at 27°C and 80% humidity. All mosquito rearing and feeding protocols were approved and in accordance with regulatory guidelines and standards set by the Institutional Animal Care and Use Committee of the University of California, Davis. For experimental treatments, laboratory reared 3–5 day old female mosquitoes were kept on water for 48 h and then allowed to feed for 30 min on reconstituted human blood meals provided through a Hemotek Insect Feeding System (Discovery Workshops).

### *P. falciparum* Culture and Mosquito Infection

For mosquito infection, cultures of *P. falciparum* strain NF54 MCB (Sanaria Inc.) were grown as previously described [83]. Mosquitoes were allowed to feed on day 15 mature gametocyte cultures diluted with human RBCs and heat-inactivated human serum with or without PKC inhibitors chelerythrine (1 µM) or Go6983 (0.05 µM). All treatments were added to the diluted *P. falciparum* culture immediately prior to blood feeding. Protocols involving the culture and handling of *P. falciparum* for mosquito feeding were approved and in accordance with regulatory guidelines and standards set by the Biological Safety Administrative Advisory Committee of the University of California, Davis. After 10 days, midguts from 50 mosquitoes with fully developed eggs (to confirm complete engorgement) from each group were dissected in PBS and stained with 0.1% mercurochrome for direct counting of *P. falciparum* oocysts. Means of oocysts per midgut in each treatment group were calculated from all dissected mosquitoes, including zeros for mosquitoes that contained no oocysts.

### *P. falciparum* Growth Assays

Aliquots of *P. falciparum* NF54 culture were synchronized 48 h prior to the assay as previously described [84] and then plated in 96 well flat bottom plates in complete RPMI 1640 with HEPES, hypoxanthine and 10% heat inactivated human serum. Parasites were treated with 10, 1, and 0.1 µM of chelerythrine or 5, 0.5, 0.05 µM of Go6983 or with an equivalent volume of DMSO diluent for 48 h in a candle jar in a 37°C incubator. Numbers of infected RBCs were determined as previously described [83].

### PCR and Quantitative Real-time PCR of mRNA Transcripts

*A. stephensi* mosquitoes were fed a blood meal and at 24 h post-blood feeding 30 mosquito midguts were dissected into RNA later (Qiagen) and homogenized using a QIAshredder column (Qiagen). RNA was extracted from homogenates using the Qiagen RNeasy mini kit per the manufacturer’s protocol. RNA samples were reverse transcribed using SuperScript® III (Invitrogen). Sample cDNAs were used to perform PCR using TaqMan Gold RT-PCR Reagents kit. Primers were designed based on AsPKC sequences using Primer Express software (Applied Biosystems). The following PKC specific primers were used: cPKC forward 5′-CACGCTTTCTTCCGTCGTAT-3′ and reverse 5′-CACAAACTCGGGGTTGAGAT-3′; PKCδ forward 5′-AACACGATGGACGAGGAGAG-3′ and reverse 5′-TGGGGTTGGTGTAGGTGAAT-3′; PKCε forward CAAAGTTCGACCACCATTCC-3′ and reverse 5′-CCAAACTCCGGATTCGTAAA-3′; PKCζ forward 5′-GTATACCGCGTTCGCTCAGT-3′ and reverse 5′-AATCACGGTCCGAGTCTAGC-3′; PKD forward 5′-GGTGTGCACCGGAAGACGCA -3′, reverse 5′-GGCAGGAGTCCCGACGACAGA-3′; and PKN forward 5′-CTGGAAGCCATCGCAATAAT-3′ and reverse 5′-TTTTCGGACGTGAACTCCtC-3′. Amplified products were electrophoretically separated through 1% agarose, stained with ethidium bromide and visualized under UV light with 1D Image Analysis Software (Kodak).

Quantitative real time-PCR was performed using Maxima SYBR Green/ROX qPCR Master Mix (Fermentas) and ABI Prism 7300 Sequence Detection System (Applied Biosystems). Amplification conditions were described previously in [50], [85]. Data from biological replicates with unique and separate groups of mosquito cells were used for statistical analysis. The data were analyzed using the 2^−ΔΔCt^ method as described [86].

### Cell Culture, PKC Kinase Activity and Luciferase Reporter Assays

ASE cells [87] were maintained as previously described [83]. For PKC kinase assays, cells were plated 48 h prior to the assay and then treated with 100 µg/ml lipopolysaccharide (LPS, *Escherichia coli* serotype 026:B6; Sigma-Aldrich) for 30 min. PKC enzymatic activity was measured using the PepTag Assay for Non-Radioactive Detection of Protein Kinase C Kit (Promega). Proteins were purified from mosquito cells using a source 15PHE 4.6/100PE 1.5ml column (GE Life Sciences) and then concentrated 100× with Centricon filters (Millipore) prior to addition of reaction master mix.

Luciferase reporter assays were performed using the Defensin, Cecropin, and Gambicin promoter-reporter plasmids as previously described [33]. At 24 h post-transfection, cells were challenged with 100 µg/ml LPS (Sigma-Aldrich), 1 µg/ml peptidoglycan (PGN, *Escherichia coli* K12; San Diego, CA) or with 150×10^6^ parasite equivalents of *P. falciparum* soluble products (PfsPs) as previously described [33]. For inhibition assays, cells were treated with the PKC inhibitors chelerythrine (1 µM; Sigma-Aldrich) or Go6983 (0.05 µM; Tocris bioscience) for 1 h prior to immune challenge. Luciferase activity was measured 24 h post-immune challenge with the Dual-Glo system (Promega).

### Preparation of *P. falciparum* Freeze/thaw Parasite Product (FTPP)

To produce *P. falciparum* freeze/thaw parasite product (FTPP) for *in vivo* experiments as previously published [88], iRBCs from day 15 parasite cultures were frozen at −80 C for 10 min and thawed at 37 C for 10 min three times. To mimic our live parasite infection protocol, FTPP was diluted with intact uninfected human RBCs and heat-inactivated human serum prior to feeding to mosquitoes. As control an equal volume of uninfected RBCs was similarly frozen and thawed and fed to mosquitoes.

### Functional Assay of Midgut Permeability in *A. stephensi*

Laboratory reared 3–5 d old female mosquitoes were kept on water for 48 h and then allowed to feed for 30 min on reconstituted human blood meals with 1×10^6^ fluorescent beads/ml (3.0–3.4 µm, Spherotech). Non-blood fed mosquitoes were removed immediately after feeding. At 72 h post-blood feeding, three whole mosquitoes or three dissected midguts were placed in PBS, pulse sonicated, filtered (35 µm nylon mesh) and analyzed by flow cytometry. Data acquisition was performed with a FACScan flow cytometer (BD Biosciences) and analysis was conducted using FlowJo software (version 6.4.1; Tree Star). The number of beads per three midguts was quantified and subtracted from each analyzed sample of three whole mosquitoes to remove the contribution of beads remaining in the midgut to whole body bead counts.

### Immunoblotting

Protein extracts were prepared, separated, and transferred to membranes as previously described [33]. Membranes were blocked in 5% nonfat dry milk (w/v) in Tris-buffered saline (pH 7.0) containing 0.1% Triton-100 (TBS-T) for 1 h at room temperature. Membranes were incubated at 4°C overnight with the following: 1∶10,000 mouse monoclonal anti-diphosphorylated ERK1/2 (p-ERK) (Sigma-Aldrich), 1∶1000 anti-phospho-FOXO1/FOXO3a (p-FOXO) (Cell Signaling), or 1∶10,000 anti-GAPDH (Sigma-Aldrich) antibody in 5% nonfat dry milk in TBS-T. Membranes were washed 3 times, 5 min each, and incubated with a 1∶2,000 (p-FOXO) or 1∶20,000 (GAPDH) dilution of horseradish peroxidase-conjugated goat anti-rabbit IgG (Biosource International) or with 1∶20,000 (p-ERK) HRP-conjugated rabbit anti-mouse IgG (Pierce) at 4°C overnight. To reveal antibody-bound proteins, membranes were incubated with SuperSignal West Dura chemiluminescent reagent for 5 m and visualized using the Kodak Image Station 4000MM Pro and Carestream Molecular Imaging software (Carestream Health). Levels of phospho-proteins in each treatment were first normalized to total protein levels as determined by GAPDH and then to the appropriate control group.

### Statistical Analyses

For mosquito infection studies, data from three independent experiments with separate cohorts of mosquitoes were analyzed for the main effects of experiment and treatment. These data were not normally distributed and, therefore, analyzed using the Kruskal–Wallis test and Dunn’s post-test (alpha = 0.05). Differences in prevalence of infection (the presence of at least one oocyst in a dissected mosquito) were determined by chi-square test (alpha = 0.05). For all other data sets, significance was determined by Student’s t-test (alpha = 0.05).

## Supporting Information

Figure S1 **Detection of phosphorylated (p-) PKCζ and PKD proteins in** ***A. stephensi*** **and** ***A. gambiae*** **cell lysates.** (A) Homology among *H. sapiens*, *A. gambiae* and *A. stephensi* for amino acid sequences recognized by antibodies to human phosphorylated PKCζ/λ (Cell signaling 9378) and PKD (Invitrogen 44961G; arrows indicate phosphorylation sites). (B) Representative western blots of protein lysates from untreated immortalized ASE (*A. stephensi*, n = 2) and 4a3B (*A. gambiae,* n = 3) cells probed with human phospho-PKCζ and phospho-PKD antibodies. GAPDH detection was used as an indication of protein loading.
(TIF)

Figure S2 **Midpoint-rooted Bayesian tree based on analysis of the UNFILT dataset.** Node support values represent Bayesian posterior probabilities (values below 0.85 are not shown). Groupings of specific PKC gene family members are marked. Accession numbers of all sequences used for this analysis are listed in Table S1.
(TIF)

Figure S3 **Exon-intron organization of** ***A. gambiae*** **and** ***A. stephensi*** **PKC genes.** (A) The exon-intron structure of *A. gambiae* cPKC, PKCδ, PKCε, PKCζ, PKD, PKN and (B) the exon-intron structure of *A. stephensi* PKCδ, PKCε, PKCζ, PKD, PKN. The exon-intron structure for *A. stephensi* cPKC could not be determined due to gaps in the coding sequence. Black boxes denote exons.
(TIF)

Figure S4 **Inhibition of PKC activation decreases ERK and FOXO phosphorylation in** ***A. stephensi*** **midgut tissues following blood feeding with parasite antigen.** Midgut tissue from *A. stephensi* fed blood meals containing *P. falciparum* freeze/thaw parasite products (FTPP) in the presence or absence of PKC inhibitors were dissected and processed for western blot analysis as previously described in [33]. (A) Representative western blots of ERK and FOXO phosphorylation at 30 m post blood feeding by a single cohort of *A. stephensi*. (B) Graph of fold changes calculated by dividing GAPDH-normalized phospho-protein levels in FTPP+PKC inhibitor-fed midgut tissues with phospho-protein levels in matched controls (FTPP alone).
(TIF)

Table S1 Accession numbers of PKC sequences utilized for the phylogeny in Figures 2 and S2.
(TIF)
