# Supplementary figures and images for: Protein Kinase C-Dependent Signaling Controls the Midgut Epithelial Barrier to Malaria Parasite Infection in Anopheline Mosquitoes
Source: PLoS One. 2013 Oct 11;8(10):e76535. doi: 10.1371/journal.pone.0076535 (PMC3795702; doi:10.1371/journal.pone.0076535)

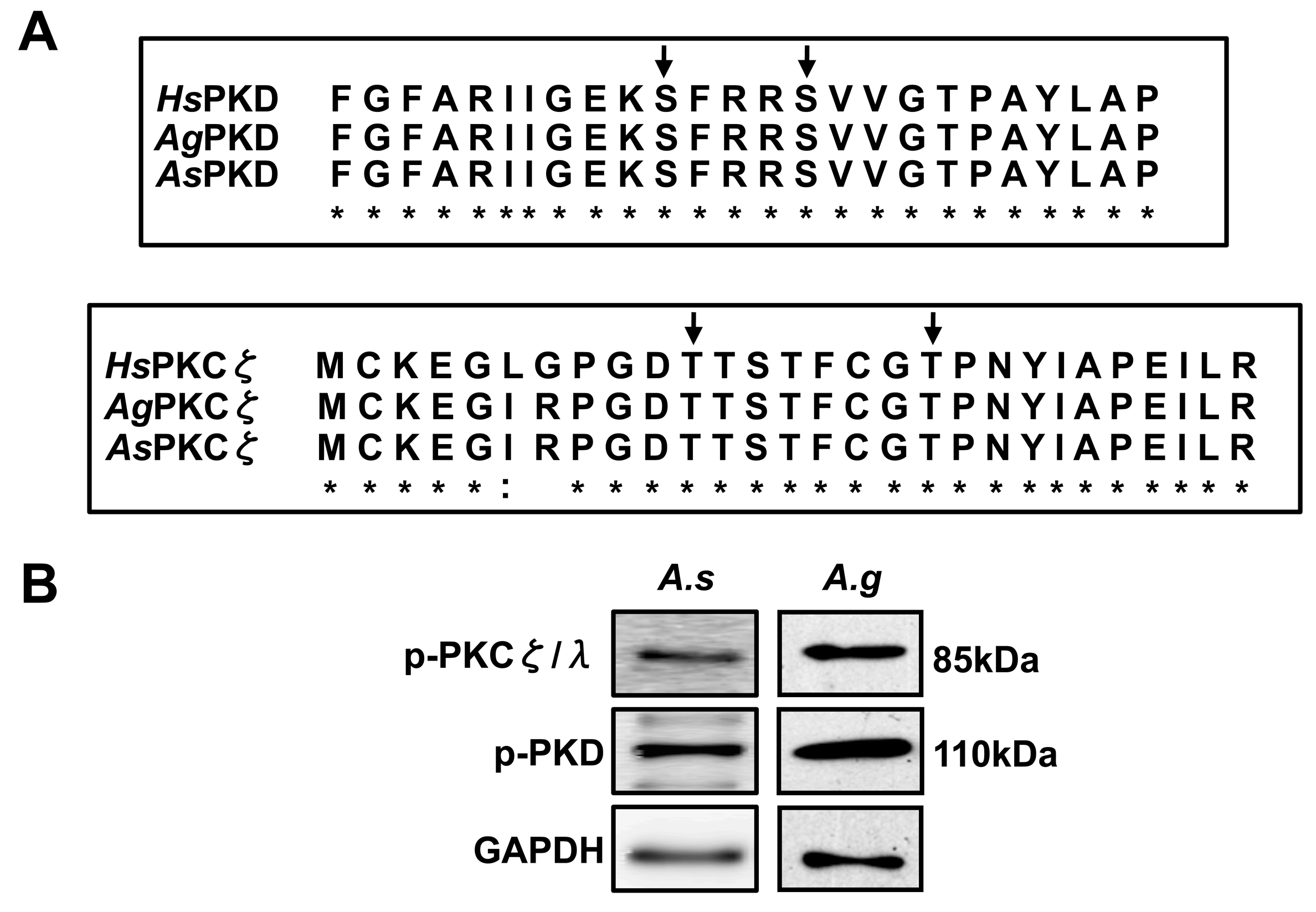

Supplement: Figure S1 — Detection of phosphorylated (p-) PKCζ and PKD proteins in A. stephensi and A. gambiae cell lysates. (A) Homology among H. sapiens, A. gambiae and A. stephensi for amino acid sequences recognized by antibodies to human phosphorylated PKCζ/λ (Cell signaling 9378) and PKD (Invitrogen 44961G; arrows indicate phosphorylation sites). (B) Representative western blots of protein lysates from untreated immortalized ASE (A. stephensi, n = 2) and 4a3B (A. gambiae, n = 3) cells probed with human phospho-PKCζ and phospho-PKD antibodies. GAPDH detection was used as an indication of protein loading. (TIF) [file pone.0076535.s001.tif]

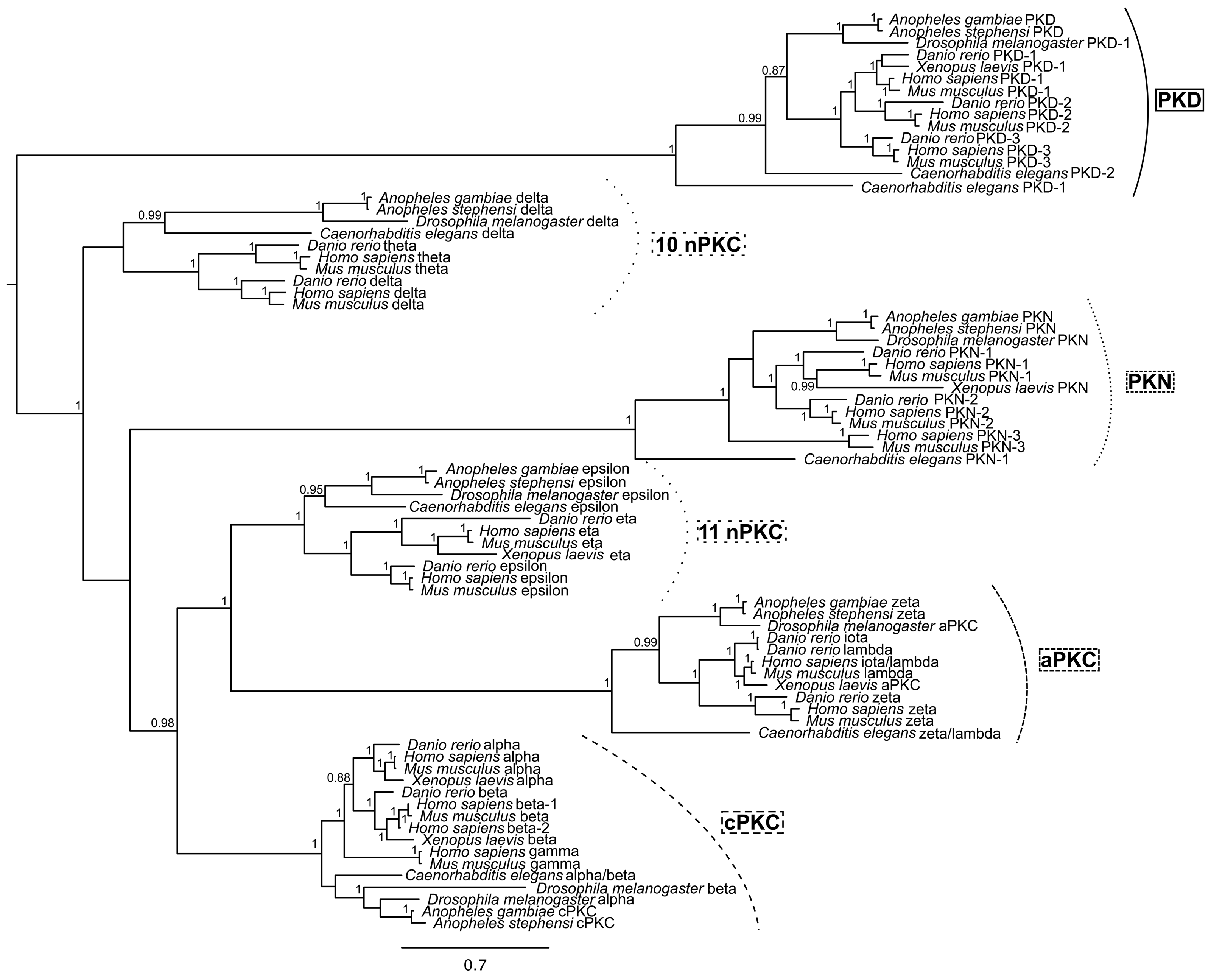

Supplement: Figure S2 — Midpoint-rooted Bayesian tree based on analysis of the UNFILT dataset. Node support values represent Bayesian posterior probabilities (values below 0.85 are not shown). Groupings of specific PKC gene family members are marked. Accession numbers of all sequences used for this analysis are listed in Table S1. (TIF) [file pone.0076535.s002.tif]

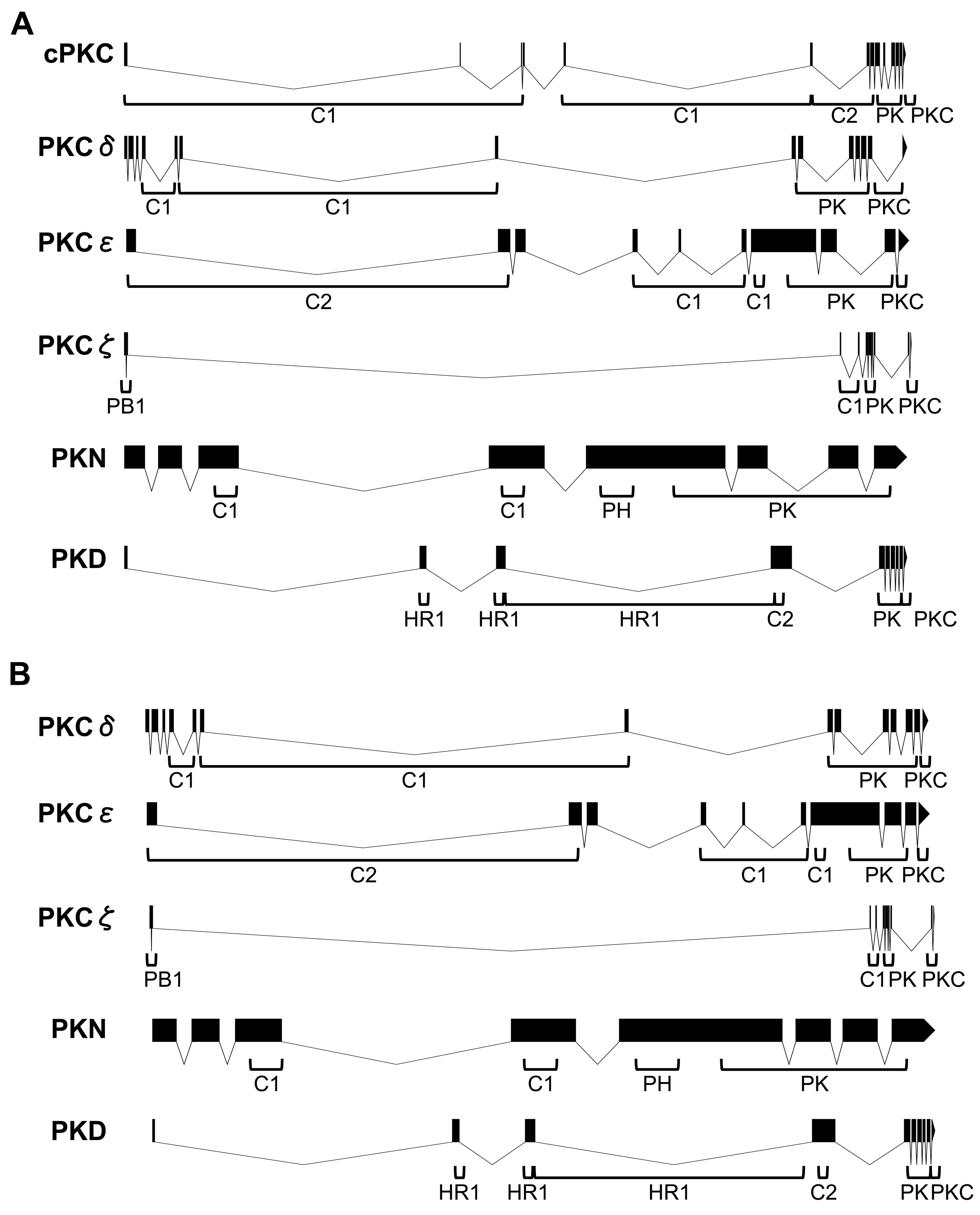

Supplement: Figure S3 — Exon-intron organization of A. gambiae and A. stephensi PKC genes. (A) The exon-intron structure of A. gambiae cPKC, PKCδ, PKCε, PKCζ, PKD, PKN and (B) the exon-intron structure of A. stephensi PKCδ, PKCε, PKCζ, PKD, PKN. The exon-intron structure for A. stephensi cPKC could not be determined due to gaps in the coding sequence. Black boxes denote exons. (TIF) [file pone.0076535.s003.tif]

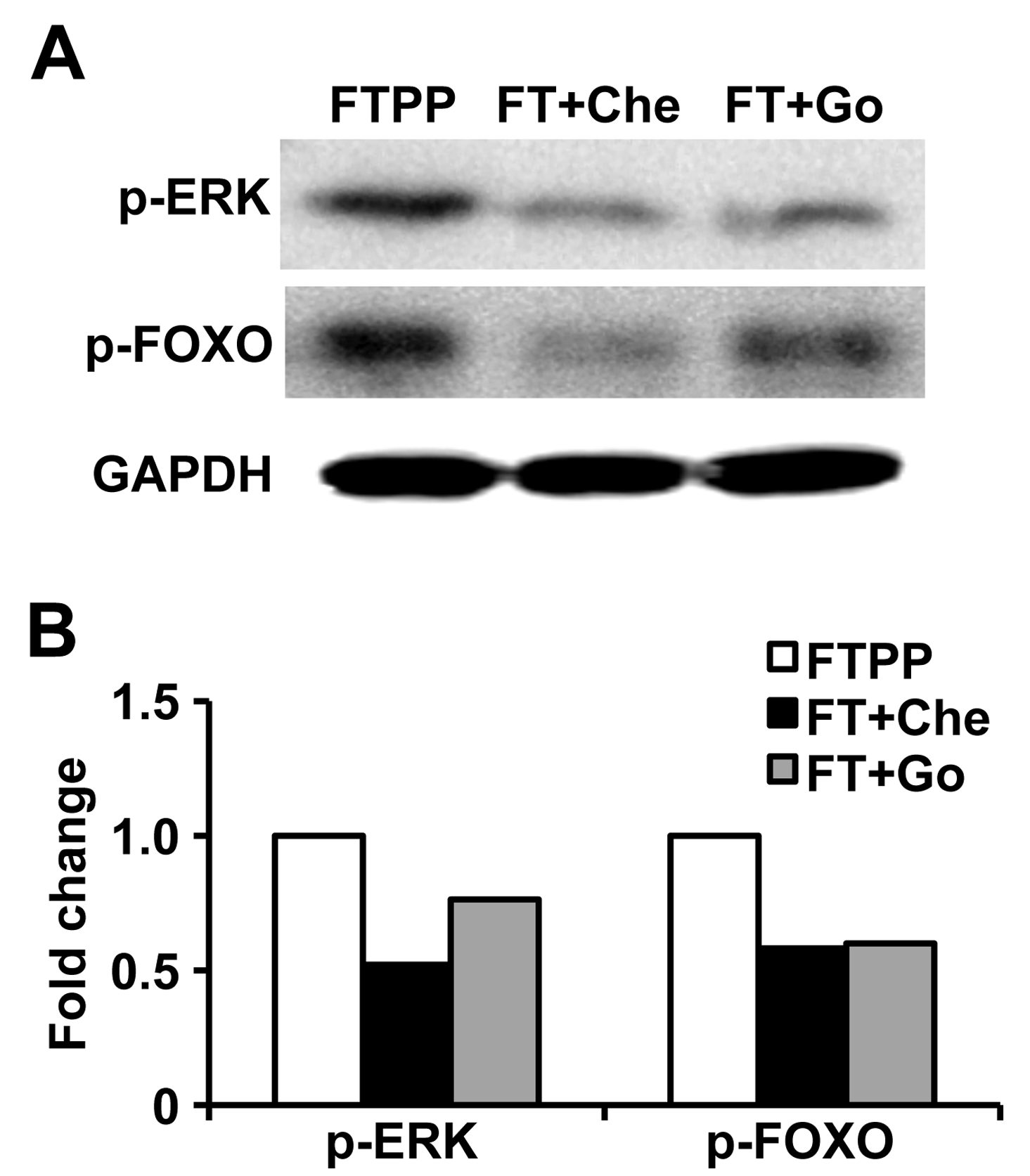

Supplement: Figure S4 — Inhibition of PKC activation decreases ERK and FOXO phosphorylation in A. stephensi midgut tissues following blood feeding with parasite antigen. Midgut tissue from A. stephensi fed blood meals containing P. falciparum freeze/thaw parasite products (FTPP) in the presence or absence of PKC inhibitors were dissected and processed for western blot analysis as previously described in [33]. (A) Representative western blots of ERK and FOXO phosphorylation at 30 m post blood feeding by a single cohort of A. stephensi. (B) Graph of fold changes calculated by dividing GAPDH-normalized phospho-protein levels in FTPP+PKC inhibitor-fed midgut tissues with phospho-protein levels in matched controls (FTPP alone). (TIF) [file pone.0076535.s004.tif]

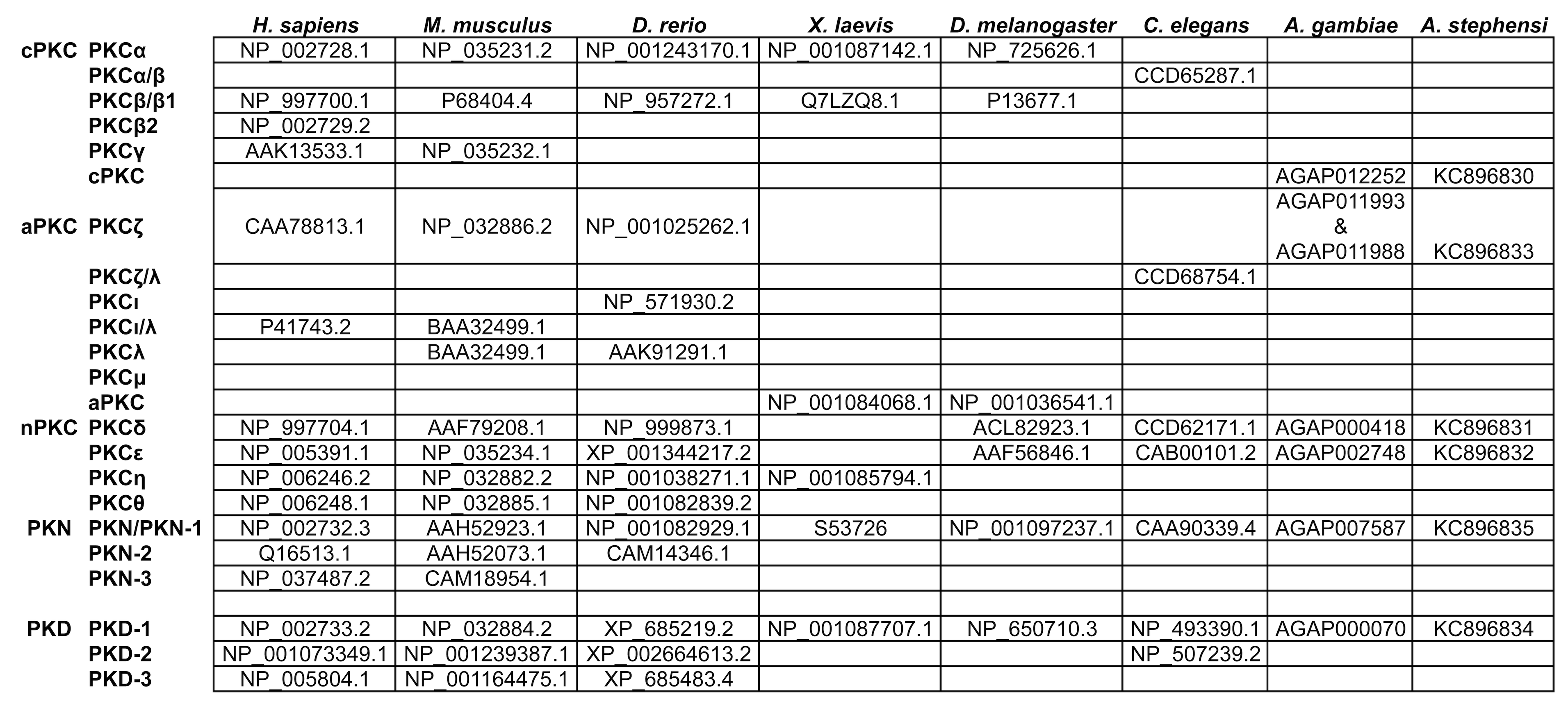

Supplement: Table S1 — Accession numbers of PKC sequences utilized for the phylogeny in Figures 2 and S2. (TIF) [file pone.0076535.s005.tif]
